# 

**Published:** 1961-04

**Authors:** 


MEDICAL JOURNAL OF THE SOUTH-WEST

*(Incorporating the Bristol Medico-Chirurgical Journal)*

Established 1883

VOL. 76 (ii) April 1961 NO. 280

### Contents

| | | |
|---|---|---|
| CAVE RESCUE | *Dr. Oliver C. Lloyd* | 37 |
| SUBARACHNOID HAEMORRHAGE | *H. Lovell Hoffman* | 50 |
| THE UNSTABLE CERVICAL DISC | *D. M. Jones* | 57 |
| THE SKELETON IN THE PIT | *G. Stewart Smith* | 61 |
| CLONORCHIASIS | *Clinical Pathological Conference* | 65 |
| NOTES AND NEWS | | 71 |
| APPOINTMENTS | | 71 |

*PUBLISHED QUARTERLY*

*ANNUAL SUBSCRIPTION 16/- POST FREE*

PUBLISHED UNDER THE AUSPICES OF THE
RISTOL MEDICO-CHIRURGICAL SOCIETY

### *Editorial Committee*

Dr. J. E. Cates, Department of Medicine, Bristol Royal Infirmary. *Editor.*
Dr. N. J. Brown, Southmead Hospital, Bristol. *Assistant Editor.*
Dr. A. B. Raper, Department of Pathology, Bristol Royal Infirmary. *Assistant Editor.*
Dr. J. Macrae, Ham Green Hospital.
Dr. R. C. Wofinden, Central Clinic, Bristol.
Mr. A. E. S. Roberts, Medical Library, University of Bristol. *Secretary.*

*Local Correspondents*

*Bath* . . Mr. A. D. Bateman, 3 The Circus, Bath.
*Exeter* . . Dr. L. N. Jackson, Mount Jocelyn, Crediton, Devon.
*Gloucester* . Dr. R. F. Jarrett, 9 College Green, Gloucester.
*Plymouth* . Dr. C. R. Croft, Lexden, Hartley Av., Plymouth.
*Taunton* . Dr. M. McAlley, 1 The Crescent, Taunton.
*Torquay* . Dr. A. Robinson Thomas, 20 Devon Square, Newton Abbot, Devon.
*Truro* . . Dr. F. D. M. Hocking, St. Andrews, Perranporth, Cornwall.
*Yeovil* . . Mr. J. A. Vere Nicoll, Knapp House, Yeovil, Somerset.

### NOTICE TO CONTRIBUTORS

Original articles are invited, provided they have not been published elsewhere. Notes of forthcoming events, meetings held, degrees conferred, staff appointments, and other local news are welcomed. The editorial committee reserves the right to make literary corrections.

Illustrations should always be supplied ready for reproduction. All re-touchings or other operations required will be charged to the author. Line drawings should be drawn in Indian ink. We regret that we must ask authors to pay for making blocks, if this is necessary.

J. W. ARROWSMITH LTD., WINTERSTOKE ROAD,
BRISTOL.

### Notes and News

As a tribute to Emeritus Professor R. J. A. Berry, late Professor of Anatomy and Dean of the Faculty of Medicine at Melbourne University, the university is to name the main anatomical lecture theatre which he designed and created "The Berry Theatre".

Dr. M. B. Lennard has been awarded a Nuffield Travelling Fellowship for General Practitioners to enable him to visit Canada and United States to study social and environmental factors in both undergraduate and post graduate training in medicine.

### Examination Results

UNIVERSITY OF BRISTOL

*M.D.:* M. S. Dunhill

*M.B., Ch.B.:*

L. M. Ajabor

Frances N. Burton

Belinda R. Davies

W. O. Gbajumo

D. D. Kennedy

W. T. Morris

J. J. Nicholas

G. H. Robb

H. Ruhomaully

R. M. Sykes

### Appointments

*UNITED BRISTOL HOSPITALS*
*JULY TO DECEMBER, 1960*

| *Name* | *Appointment* | *Formerly* |
|---|---|---|
| CARTER, Miss S .J., M.B., B.S., M.R.C.P., D.M.R.D. | Senior Registrar in Radiology | Registrar in Radiology, U.B.H |
| CRAIG, J. K., M.B., CH.B., F.R.C.S., F.R.C.S.E. | Senior Registrar in Obstetrics and Gynaecology | Senior Registrar, Maternity Hospital, Leeds. |
| WOODHOUSE, D. F., B.M., B.CH., D.O. | Senior Registrar in Ophthalmology. | Registrar, Birmingham and Midland Eye Hospital. |
| RUSSELL, Miss P. M., M.D., CH.B., M.R.C.O.G., D.C.H. | Registrar in Obstetrics and Gynaecology. | Demonstrator in Anatomy, University of Bristol. |
| MACRAE, W. R., M.B., CH.B. | Registrar in Anaesthetics | S.H.O. in Anaesthetics, U.B.H |
| THORPE, M. H., M.B., CH.B. | Registrar in Anaesthetics. | S.H.O. in Anaesthetics, United Sheffield Hospitals. |
| CLARK, P. A., M.B., B.S. | Registrar in Pathology. | Junior Clinical Pathologist U.B.H. |
| WILLS, M. R., M.B., CH.B. | Registrar in Chemical Pathology. | Registrar in Pathology, U.B.H. |
| FAIRBURN, A. C., M.B., B.S., M.R.C.P., D.C.H., D.P.M. | Senior Registrar in Paediatrics. | Registrar, Maudsley and Bethlem Royal Hospitals. |
| EADES, Miss S., M.B., B.S. | Registrar in Paediatrics. | Registrar, South Devon and E. Cornwall Hospitals. |
| GIBBON, R. H., M.B., B.CH. | Registrar in Radiology. | Part-time Registrar in Radiology, U.B.H. |
| PENRY, J. B., M.B., B.CH. | Registrar in Radiology. | Part-time Registrar in Radiology, U.B.H. |

Dr. M. Jackman, Registrar in Radiology, has been appointed to a research post, with Registrar status, in the United Bristol Hospitals for six months from 2nd January, 1961.

### *SOUTH WESTERN REGIONAL HOSPITAL BOARD*
### *JULY TO DECEMBER*, 1960

| *Name* | *Appointment* | *Formerly* |
|---|---|---|
| | ***BRISTOL*** | |
| Routledge, R. T., F.R.C.S. | Consultant Plastic Surgeon, Bristol Clinical Area. | Senior Registrar in Plastic Surgery, Frenchay Hospital and U.B.H. |
| Flood, R. A., M.B., CH.B. D.P.M.<br>Bird, R. L., M.B., B.S., D.P.M. | Senior Registrars in Psychiatry, Glenside and Barrow Hospitals. | Registrars in Psychiatry, Glenside and Barrow Hospitals |
| Williams, D. P. C., F.R.C.S., D.L.O. | Senior Registrar in E.N.T. Bristol Clinical Area. | E.N.T. Registrar, Bristol Clinical Area. |
| Benney, W. E., B.SC., M.B., CH.B. | Medical Registrar, Cossham-Frenchay Group. | Locum in General Practice. |
| O'Sullivan, Patricia, M., M.B.B.S. | Registrar in Pathology, Frenchay Hospital. | Registrar in Pathology, General Infirmary, Leeds. |
| Trapnell, J. E., M.A., M.B.B.CHIR. | Surgical Registrar, Cossham-Frenchay Group. | R.M.O., Homoeopathic Hospital, Bristol. |
| Hederman, Helen, M., M.B.B.CH., D.A. | Anaesthetic Registrar, Southmead Hospital. | S.H.O. in Anaesthetics, South Teeside Group of Hospitals |
| Andrews, Joan, M.B.B.S., D.OBST.R.C.O.G. | Registrar in Obsterics and Gynaecology, Southmead Hospital. | St. David's Hospital, Cardiff. |
| Chowdbury, S. R., M.B.B.S. | Surgical Registrar, Weston-super-Mare General Hospital. | Locum Surgical Registrar, Grantham and Kesteven Hospital. |
| Foster, A. R., B.SC., M.R.C.P.E., D.P.M. | Assistant Psychiatrist (S.H.M.O. grade), Glenside and Barrow Hospitals. | Senior Psychiatric Registrar, Glenside and Barrow Hospitals. |
| Gibbs, Irene, E., M.B., CH.B. | Clinical Assistant in Paediatrics, Southmead Hospital Group. | Locum—General Practice. |
| Hill, B. W., M.B., CH.B.,<br>Wethered, R. R., B.M., B.CH. | Clinical Assistants in Dermatology, Southmead and Cossham Hospitals. | re-appointed. |
| Azariah, R., M.B., B.S. | Registrar in Neurological Surgery, Frenchay Hospital. | Casualty Officer, Croydon General Hospital. |
| Robertson, D. A. S., M.B., CH.B., D.OBST., R.C.O.G. | Registrar in Psychiatry, Glenside and Barrow Hospitals. | S.H.O. in Psychiatry, Glenside and Barrow Hospitals. |
| Midwinter, R. E., B.SC., M.B., CH.B., D.C.H. | Registrar in Paediatrics, Southmead Hospital. | S.H.O. in Paediatrics, Southmead Hospital. |
| Manalo, G., M.D. | Registrar in Thoracic Surgery, Frenchay Hospital. | Locum Registrar in Thoracic Surgery, Frenchay Hospital. |
| | ***BATH*** | |
| McNulty, M.B., B.CH., B.A.O., D.M.R.D., F.F.R. | Consultant Radiologist, Bath Clinical Area. | Senior Registrar, Manchester Royal Infirmary. |
| Anderson, J. C., M.CH.ORTH., F.R.C.S. | Senior Registrar in Orthopaedic and Traumatic Surgery, Bath Clinical Area.<br>Surgical Registrar, Bath Group of Hospitals. | Orthopaedic Consultant, Cape Town, S. Africa. |
| MacMath, I. F., B.SC., M.B., CH.B., D.OBST.R.C.O.G. | Clinical Assistant in Obstetrics and Gynaecology, Trowbridge and Bradford-on-Avon Hospitals. | re-appointed. |
| Stewart, D. J., M.B., CH.B. | Clinical Assistant in Orthopaedic Surgery, Bath. | re-appointed. |
| Macneil, P. R., F.R.C.S. | Surgical Registrar to the Bath Group of Hospitals. | Locum Senior Registrar, West Middlesex Hospital. |
| | ***SOUTH SOMERSET*** | |
| Bahl, B.R., F.R.C.S. | Surgical Registrar, Taunton and Somerset Hospital. | Surgical Registrar, Friarage Hospital, North Allerton. |

| | | |
|---|---|---|
| | *NORTH GLOUCESTERSHIRE* | |
| MOFFATT, W. H., M.B., B.CH., B.A.O., M.R.C.P.I. | Assistant Geriastrician, (S.H.M.O. grade), North Gloucestershire Clinical Area. | Senior Registrar, Geriatric Unit, Belfast City Hospital. |
| SEWELL, I. A., M.B., B.S. | Surgical Registrar, Cheltenham General Hospital. | Casualty Surgical Registrar, The Middlesex Hospital, London. |
| CADZOW, W. H., M.B.B.S. | Surgical Registrar, Gloucestershire Royal Hospital. | Locum Surgical Registrar, Gloucestershire Royal Hospital. |
| HOLDING, M. F., M.B., B.CH., D.OBST.R.C.O.G. | Medical Registrar, Gloucestershire Royal Hospital. | Medical Registrar, West Wales General Hospital. |
| FOLEY, P. C., M.B., B.CH. | Medical Registrar, Cheltenham General Hospital. | S.H.O. in Medicine, Ryhope General Hospital. |
| | *SOUTH SOMERSET* | |
| BARDFIELD, A. M., M.B., CH.B., M.R.C.P., D.P.M. | Consultant Psychiatrist, South Somerset Clinical Area. | S.H.M.O., Glenside and Barrow Hospitals. |
| BUSU, S. K., M.B., D.A. | Anaesthetic Registrar, Taunton and Somerset Hospital. | Chief Anaesthetist, Wanless Tuberculosis Sanatorium, Bombay. |
| EGGLESTON, M.D., M.B., CH.B. | Clinical Assistant in Geriatrics, Taunton. | |
| | *DEVON AND CORNWALL* | |
| HILLIS, B. R., M.D., CH.B., F.R.F.P.S., M.R.C.P.E. | Consultant General Physician, North Devon. | Senior Registrar and Lecturer, University of Glasgow. |
| BURLEY, M. A., F.D.S., D.ORTH. | Consultant Othodontist, Devon and Cornwall. | Consultant Orthodontist, United Sheffield Hospitals. |
| ROBINS, R. H. C., F.R.C.S. | Consultant Orthopaedic Surgeon, West Cornwall Clinical Area. | Senior Orthopaedic Registrar, Exeter Clinical Area. |
| MONTGOMERY, J. N., M.D., M.R.C.P., D.C.H. | Consultant Paediatrician, Plymouth Clinical Area. | Senior Registrar, Paediatric Department, St. Mary's Hospital, London. |
| HASS, L., M.A., M.D., B.CH., M.R.C.P., D.C.H. | Consultant Paediatrician, Torquay Area. | Senior Registrar, The Queen Elizabeth Hospital for Children, London. |
| JOHNSTON, W., M.B., CH.B., D.P.M. | Consultant in Mental Deficiency, Royal Western Counties Group of Hospitals. | Deputy Physician Superintendent, Royal Scottish National Institution, Larbert. |
| WHITEHEAD, S. T., M.B., CH.B. | Surgical Registrar, Torbay/Newton Abbot Hospitals. | Surgical Registrar, Croydon General Hospital. |
| SCOTT, P. A., M.D., C.M. | Orthopaedic Registrar, Mount Gold Hospital, Plymouth. | Senior Interne, Department of Surgery, Kingston General Hospital, Canada. |
| GAUDIN, D. A., B.M., M.D. | Orthopaedic Registrar, Mount Gold Hospital, Plymouth | Orthopaedic Surgery, Shaughnessy Hospital, Vancouver, Canada. |
| AZZOPARDI, S. J., M.D. | Paediatric Registrar, South Devon and East Cornwall Hospital, Plymouth. | Paediatric Registrar, West Middlesex Hospital. |
| COLE, G. VINE, M.R.C.S., L.R.C.P., D.O. | Ophthalmologist (S.H.M.O.) Plymouth Clinical Area. | Senior Ophthalmic Registrar, Cardiff Royal Infirmary. |
| MANSFIELD, G. C., M.B.CH.B., D.OBST.R.C.O.G. | General Practitioner Anaesthetist, Totnes and Brixham Hospitals. | G.P. Anaesthetist, Paignton and South Hams Hospitals, Kingsbridge. |
| FELTON, D. J., M.B., CH.B. | Registrar in Obstetrics and Gynaecology, Royal Devon and Exeter Hospital. | S.H.O. in Obstetrics, Saint Mary's Hospitals, Manchester. |
| ADAMS, J. A., F.R.C.S., M.CH.(ORTH.) | Senior Registrar in Orthopaedic and Traumatic Surgery, Exeter Clinical Area. | Locum Orthopaedic Senior Registrar, King's College Hospital, London. |
| WYNNE-DAVIES, RUTH, F.R.C.S. | Research Registrar in Orthopaedics, Princess Elizabeth Orthopaedic Hospital, Exeter. | S.H.O., Royal Salop Infirmary, Shrewsbury. |

| | | |
|---|---|---|
| HELLON, C. P., M.B., CH.B. | Registrar in Psychiatry, Moorhaven Hospital, Ivybridge. | S.H.O. in Psychiatry, Southern General Hospital, Glasgow. |
| HONSY, S. H., F.R.C.S.E., D.S. | Surgical Registrar, Royal Cornwall Infirmary, Truro. | |
| SAHA, K. K., B.SC., M.B., B.S. | Registrar in Orthopaedic and Truamatic Surgery, Royal Cornwall Infirmary, Truro. | Locum Orthopaedic Registrar, Royal Sea Bathing Hospital, Margate, Kent. |

